# ALBI-SII grade: a novel prognostic indicator superior to Child–Pugh score for long-term survival in hepatocellular carcinoma and its nomogram construction and validation

**DOI:** 10.3389/fonc.2026.1781745

**Published:** 2026-04-24

**Authors:** Can Li, Xiaopeng Yu, Renyi Yang, Puhua Zeng

**Affiliations:** 1 Hunan Provincial Hospital of Integrated Traditional Chinese and Western Medicine, Hunan University of Chinese Medicine, Changsha, Hunan, China; 2 Institute of Traditional Chinese Medicine Oncology, Hunan Academy of Chinese Medicine, Changsha, Hunan, China

**Keywords:** ALBI–SII grade, hepatocellular carcinoma, nomogram, prognosis, systemic immune-inflammation index

## Abstract

**Introduction:**

Prognostic heterogeneity is common in patients with hepatocellular carcinoma (HCC) within the same clinical stage. While the albumin–bilirubin (ALBI) score and systemic immune-inflammation index (SII) have individual prognostic value, their combined significance remains unclear. We aimed to develop a novel ALBI-SII grade and a prognostic nomogram to refine risk stratification.

**Methods:**

This retrospective study included 210 patients with HCC diagnosed between 2013 and 2021. A novel ALBI–SII grade was constructed by integrating ALBI and SII. Overall survival (OS) was analyzed using Kaplan–Meier methods and Cox regression based on a multiple imputation dataset. A nomogram incorporating ALBI–SII grade and China Liver Cancer (CNLC) staging was developed and validated using time-dependent ROC curves, DeLong’s test, and decision curve analysis (DCA).

**Results:**

The ALBI–SII grade stratified patients into three distinct risk groups (Median OS: Grade 0, 639 days; Grade 1, 310 days; Grade 2, 148 days; P < 0.001). In multivariate analysis, ALBI-SII grade remained an independent prognostic factor (Grade 2 vs. 0: HR = 2.576, 95% CI: 1.654–4.012, P < 0.001). The 5-year AUC of ALBI-SII was 0.735, which was statistically comparable to that of CNLC staging (AUC = 0.724; DeLong’s P > 0.05). The nomogram demonstrated robust discrimination (C-index: 0.695) and favorable clinical net benefit.

**Conclusion:**

The ALBI–SII grade is an objective and practical prognostic indicator for HCC. A nomogram integrating ALBI–SII and CNLC staging provides reliable survival prediction, particularly for long-term outcomes.

## 1 Introduction

Hepatocellular carcinoma (HCC) remains the third leading cause of cancer-related mortality worldwide, with particularly high incidence and mortality rates in East Asia (1). In China, the China Liver Cancer (CNLC) staging system, which integrates tumor burden, liver function, and performance status, is widely used to guide clinical decision-making (2). However, substantial heterogeneity in survival outcomes is frequently observed among patients within the same CNLC staging, indicating that the current prognostic stratification systems may be insufficient for precise risk assessment.

The prognosis of HCC is influenced not only by tumor-related factors, such as tumor size and vascular invasion, but also by underlying hepatic reserve and host immune-inflammatory status. The Child–Pugh (CP) classification has long been considered the standard tool for evaluating liver function (3). Nevertheless, CP classification has notable limitations, including the subjectivity involved in assessing ascites and hepatic encephalopathy, as well as the broad cutoff ranges for albumin and bilirubin, which often result in clustering of patients within CP grade A and reduced prognostic discrimination (4).

To address these limitations, Johnson et al. proposed the albumin–bilirubin (ALBI) score, an objective liver function assessment model based solely on serum albumin and bilirubin levels (4). Accumulating evidence suggests that the ALBI grade provides more refined prognostic stratification than CP classification across different disease stages and treatment modalities. In parallel, cancer-related inflammation has been recognized as a key driver of tumor progression, angiogenesis, and metastasis (5). The systemic immune-inflammation index (SII), derived from neutrophil, platelet, and lymphocyte counts, reflects the balance between pro-tumor inflammatory activity and anti-tumor immune responses and has been reported as a prognostic biomarker in HCC (6).

Although the prognostic value of ALBI and SII has been independently validated, data regarding their combined prognostic significance remain limited, particularly within the CNLC staging framework and with respect to long-term survival outcomes (7). Therefore, the present study aimed to evaluate the prognostic value of a novel ALBI–SII grade in patients with HCC and to develop and validate a nomogram integrating ALBI–SII and CNLC staging for individualized overall survival prediction.

## 2 Materials and methods

### 2.1 Study population

This retrospective study analyzed 210 patients diagnosed with and treated for HCC at Hunan Provincial Hospital of Integrated Traditional Chinese and Western Medicine between 2013 and 2021.

The inclusion criteria were as follows:

1. HCC diagnosis confirmed by histopathology or imaging criteria;
2. complete clinical and follow-up data available.

The exclusion criteria were as follows:

1. presence of other concurrent malignant tumors;
2. history of anti-tumor treatment prior to HCC diagnosis;
3. severe infection or hematological disorders that could affect peripheral blood counts.

### 2.2 Variable definition and calculation

Baseline demographic characteristics, laboratory parameters, and tumor-related features were collected for all patients.

The ALBI score was calculated using the formula derived from the study (4): $\text{ALBI}\;=(\log_{10}\text{Bilirubin}[\text{μmol}/\text{L}]\times 0.66)+(\text{Albumin}[\text{g}/\text{L}]\times -0.085)$. Based on the calculated scores, patients were stratified into low-risk (score ≤ -2.60) and high-risk (score > -2.60) groups to generate a binary ALBI variable (0 for low-risk, 1 for high-risk).

The SII was calculated based on peripheral blood cell counts using the following formula(6): $\text{SII}=\frac{\text{Platelet count}\times \text{Neutrophil count}}{\text{Lymphocyte count}}$. Using the median value of 575 as the cutoff, patients were divided into high and low SII groups.

Finally, the ALBI-SII grade was constructed by summing the binary scores of the ALBI and SII components. Patients were classified into three distinct prognostic grades based on the following explicit combination criteria:

1. ALBI-SII Grade 0 (Low Risk): Patients with both low ALBI and low SII (Score 0 + 0).
2. ALBI-SII Grade 1 (Intermediate Risk): Patients with either high ALBI and low SII, or low ALBI and high SII (Score 1 + 0 or Score 0 + 1).
3. ALBI-SII Grade 2 (High Risk): Patients with both high ALBI and high SII (Score 1 + 1).

### 2.3 Follow-up and study endpoint

The primary endpoint of this study was overall survival (OS), OS is determined from the time of diagnosis to the last follow-up (January 5, 2022). The median follow-up time was calculated using the reverse Kaplan–Meier method to avoid bias from the high mortality rate in the cohort.

### 2.4 Statistical analysis

Statistical analyses were performed using SPSS version 27 and R version 4.2.1 (survival, survminer, ggplot2, stdca.R, timeROC, stats, rms). Baseline characteristics were compared using the chi-square test. Survival curves were generated using the Kaplan–Meier method and compared with the log-rank test. Missing data for multivariate cox proportional hazards regression analyses (e.g., Hepatitis B, Liver Cirrhosis, AFP) were addressed using multiple imputation by chained equations (MICE) to minimize bias. Ten imputed datasets were generated, and the results were pooled according to Rubin’s rules. Multivariate Cox proportional hazards regression analyses were conducted to identify independent prognostic factors for OS. Nomogram construction and validation were based on the results of multivariate analysis. Time-dependent receiver operating characteristic (ROC) curves and calibration curves were used to evaluate model performance, the time-dependent Area Under the Curve (AUC) was compared using DeLong’s test. Internal validation was performed using bootstrap resampling with 5,000 iterations to assess the robustness of regression coefficients and to calculate the 95% confidence interval (CI) of the concordance index (C-index). To further evaluate the model’s robustness and generalizability, a temporal validation was performed. The entire study cohort was split into a training cohort (N = 147, diagnosed in the earlier period) and a temporal validation cohort (N = 63, diagnosed in the later period). The model established in the training cohort was then applied to the validation cohort to assess its predictive performance using the C-index. Decision curve analysis (DCA) was applied to evaluate the clinical net benefit of the model. A two-sided P value < 0.05 was considered statistically significant.

## 3 Results

### 3.1 Baseline characteristics

A total of 210 patients were included. The median follow-up time for the entire cohort was 977.5 days (interquartile range: 435.0–3102.5 days), calculated based on the censored surviving patients. According to the ALBI-SII grade, 38 patients were classified as Grade 0 (low risk), 96 as Grade 1 (intermediate risk), and 76 as Grade 2 (high risk). As shown in **Table 1**, patients with higher ALBI-SII grades tended to have higher AFP levels, higher AST levels, and more advanced CNLC staging (all P < 0.01). The median survival time decreased progressively with increasing ALBI-SII grade (P < 0.001).

**Table 1:** Basic data of HCC patients.

Characteristics	0	1	2	P value
n	38	96	76	
Age, mean ± sd	58.026 ± 14.153	57.562 ± 11.383	55.763 ± 12.519	0.542
Gender, n (%)				0.369
Female	8 (21.1%)	22 (22.9%)	11 (14.5%)	
Male	30 (78.9%)	74 (77.1%)	65 (85.5%)	
Hepatitis B, n (%)				0.707
No	7 (18.4%)	13 (13.5%)	12 (15.8%)	
Yes	23 (60.5%)	66 (68.8%)	51 (67.71%)	
Missing	8 (21.1%)	17 (17.7%)	13 (17.1%)	
ECOG PS, n (%)				< 0.001
1	0 (0%)	1 (1.0%)	0 (0%)	
2	22 (57.9%)	41 (42.7%)	15 (19.7%)	
3	14 (36.8%)	40 (41.7%)	40 (52.6%)	
4	2 (5.3%)	14 (14.6%)	21 (27.6%)	
Liver cirrhosis, n (%)				0.858
No	2 (5.3%)	3 (3.1%)	3 (3.9%)	
Yes	27 (71.1%)	67 (69.8%)	50 (65.8%)	
Missing	9 (23.7%)	26 (27.1%)	23 (30.3%)	
AST, median (IQR) (n=36, 96, 76)	39.85 (29.075, 66.85)	66.9 (38.975, 140.68)	100.05 (48.25, 179.25)	< 0.001
ALT, median (IQR) (n=37, 96, 76)	35.95 (23, 50.75)	38.25 (23.95, 67.65)	49.85 (28.775, 79.75)	0.080
AFP, median (IQR) (n=31, 83, 54)	65.48 (10.89, 263.2)	197.07 (37.63, 1210)	395.81 (46.54, 6723.8)	0.004
CNLC, n (%)				< 0.001
IA	4 (10.5%)	1 (1.0%)	0 (0%)	
IB	4 (10.5%)	4 (4.2%)	0 (0%)	
IIA	4 (10.5%)	14 (14.6%)	2 (2.6%)	
IIB	3 (7.9%)	4 (4.2%)	2 (2.6%)	
IIIA	3 (7.9%)	10 (10.4%)	4 (5.3%)	
IIIB	4 (10.5%)	14 (14.6%)	16 (21.1%)	
IV	16 (42.1%)	49 (51.0%)	52 (68.4%)	
Child-Pugh Score, n (%)				0.086
A	23 (60.5%)	53 (55.2%)	30 (39.5%)	
B	14 (36.8%)	41 (42.7%)	42 (55.3%)	
C	0 (0%)	2 (2.1%)	4 (5.3%)	
Missing	1 (2.6%)	0 (0.0%)	0 (0.0%)	
Gross Tumor Classification, n (%)				0.588
Massive	18 (47.4%)	54 (56.3%)	46 (60.5%)	
Nodular	17 (44.7%)	36 (37.5%)	23 (30.3%)	
Diffuse	3 (7.9%)	6 (6.3%)	7 (9.2%)	
Survival Time (day), median (IQR)	639 (280, 1338)	310 (175.5, 856.75)	147.5 (71.75, 318.5)	< 0.001
Treatment Modality, n (%)				0.004
Curative	13 (34.2%)	17 (17.7%)	7 (9.2%)	
Non-curative	25 (65.8%)	79 (82.3%)	69 (90.8%)	

Data are presented as median (IQR), or number (percentage) based on the original dataset to reflect the actual cohort characteristics. AFP, alpha-fetoprotein; ALBI, albumin-bilirubin; ALT, alanine aminotransferase; AST, aspartate aminotransferase; CNLC, China Liver Cancer staging; HBV, hepatitis B virus; ALBI, Albumin-Bilirubin grade;SII, systemic immune-inflammation index.

### 3.2 Prognostic value of ALBI-SII grade

**Figure 1:**
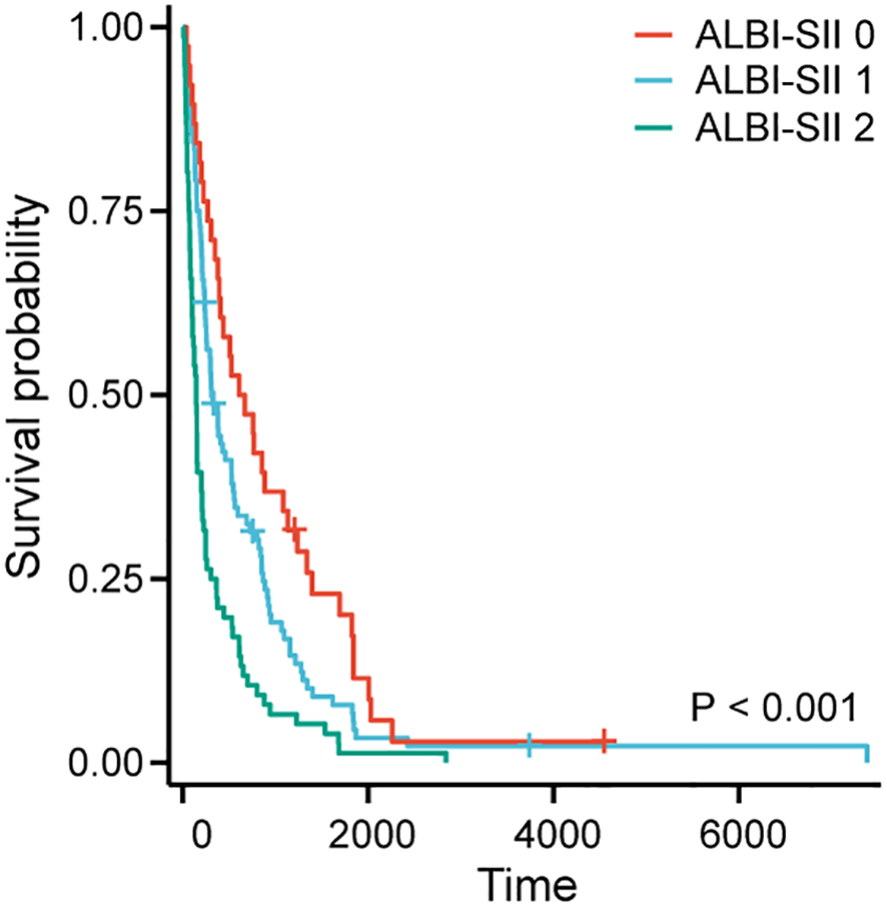
Kaplan–Meier survival curves according to ALBI–SII grade. The Kaplan–Meier curves depict the overall survival (OS) of patients stratified into three risk groups: Grade 0 (Low risk, red line), Grade 1 (Intermediate risk, blue line), and Grade 2 (High risk, green line). The survival probability decreased significantly with increasing ALBI-SII grade (P < 0.001, log-rank test). Tick marks on the curves represent censored data. ALBI, Albumin-Bilirubin grade; SII, Systemic Immune-inflammation Index.

Kaplan–Meier survival analysis demonstrated that the ALBI-SII grade effectively stratified patients with significantly different survival outcomes (**Figure 1**, P < 0.001). Patients classified as ALBI-SII Grade 0 exhibited significantly longer median OS compared with those in Grades 1 and 2. Specifically, patients with ALBI-SII Grade 0 had the longest survival, with a median OS of 639 days. In contrast, the median OS significantly decreased to 310 days for Grade 1 and 148 days for Grade 2.

In the multivariate Cox regression analysis using the original cohort, ALBI-SII grade remained an independent prognostic factor (Grade 2 vs 0: HR = 2.692, P = 0.003).To address potential bias from missing data and treatment heterogeneity, we performed a multivariate analysis based on the multiple imputation dataset (**Table 2**). The ALBI-SII grade remained a robust independent predictor of OS. Patients with ALBI-SII Grade 1 (HR = 1.661, 95% CI: 1.073–2.571, P = 0.023) and Grade 2 (HR = 2.576, 95% CI: 1.654–4.012, P < 0.001) had a significantly higher risk of mortality compared to those with Grade 0.

**Table 2:** Multivariate Cox regression analysis in the original and multiple imputation cohorts.

Characteristics	Original Cohort (n=105)		Imputed Cohort (n=210)	
	Hazard ratio (95% CI)	P value	Hazard ratio (95% CI)	P value
Gender				
Female	Reference			
Male	1.194 (0.632–2.257)	0.585	1.250 (0.849–1.840)	0.258
Age	0.998 (0.977–1.019)	0.868	0.996 (0.984–1.008)	0.488
Hepatitis B				
No	Reference			
Yes	1.125 (0.552–2.290)	0.736	1.070 (0.505–2.266)	0.859
Liver cirrhosis				
No	Reference			
Yes	1.219 (0.385–3.856)	0.736	1.070 (0.505–2.266)	0.859
AFP	1.000 (1.000 - 1.000)	0.965	1.000 (1.000–1.000)	0.924
CNLC				
IA	Reference		Reference	
IB	0.589 (0.085–4.077)	0.592	0.716 (0.218–2.351)	0.582
IIA	0.755 (0.190–3.003)	0.690	0.642 (0.227–1.814)	0.403
IIB	0.752 (0.130–4.361)	0.751	0.726 (0.223–2.366)	0.595
IIIA	0.875 (0.227–3.382)	0.847	0.787 (0.278–2.227)	0.651
IIIB	1.686 (0.444–6.399)	0.443	0.888 (0.326–2.421)	0.817
IV	2.726 (0.799–9.294)	0.109	1.944 (0.759–4.975)	0.166
Treatment Modality				
Non-curative	Reference		Reference	
Curative	0.439 (0.247–0.781)	0.005	0.493 (0.327–0.742)	<0.001
ALBI-SII				
0	Reference		Reference	
1	2.080 (1.097–3.942)	0.025	1.661 (1.073–2.571)	0.023
2	2.692 (1.407–5.152)	0.003	2.576 (1.654–4.012)	<0.001

Multivariate analysis was performed using the pooled results from the multiple imputation dataset to minimize bias caused by missing values. CI, confidence interval; HR, hazard ratio; Ref, reference category; ALBI, albumin-bilirubin; CNLC, China Liver Cancer staging; SII, systemic immune-inflammation index.

### 3.3 Predictive performance comparison: ALBI-SII versus Child–Pugh

Time-dependent ROC curve analysis revealed that the ALBI-SII grade consistently exhibited superior predictive accuracy compared with the Child–Pugh classification across multiple time points.

**Figure 2:**
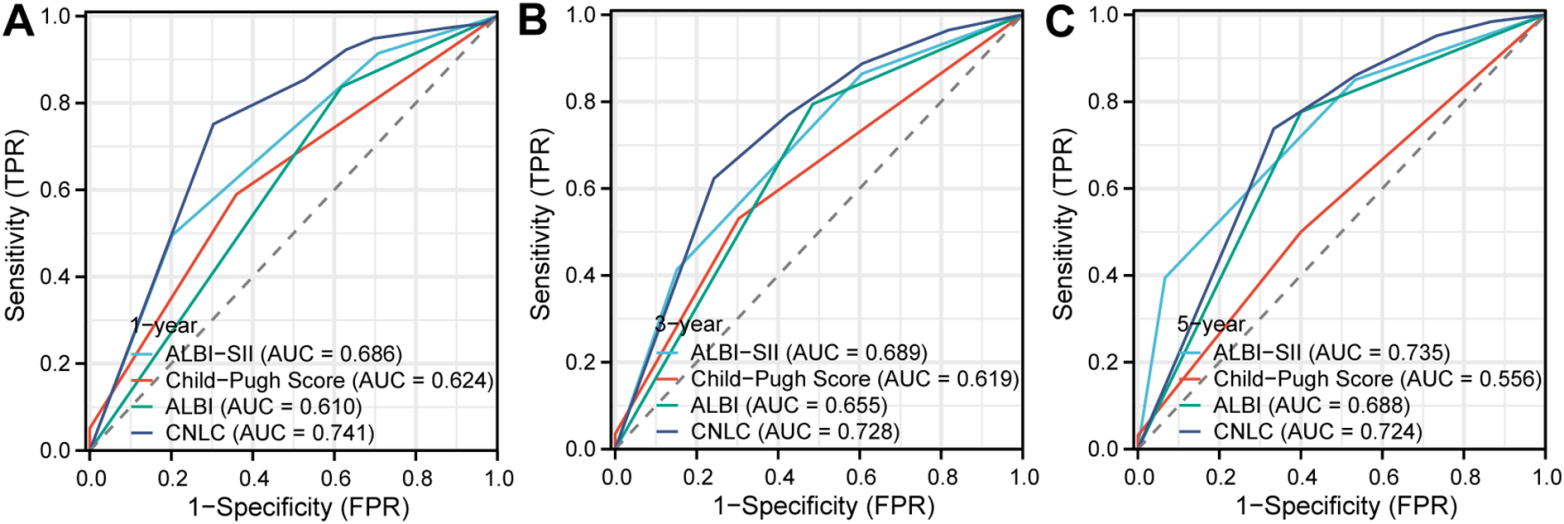
Time-dependent ROC curves for prognostic performance. Comparison of the predictive performance of ALBI-SII grade (light blue), Child-Pugh score (red), ALBI grade (green), and CNLC staging (dark blue) for predicting **(A)** 1-year, **(B)** 3-year, and **(C)** 5-year overall survival. The Area Under the Curve (AUC) for each model is listed in the respective panel. The ALBI-SII grade demonstrated superior or comparable discriminatory ability, particularly for long-term survival prediction. ROC, Receiver Operating Characteristic; AUC, Area Under the Curve; CNLC, China Liver Cancer staging; ALBI, Albumin-Bilirubin; SII, systemic immune-inflammation index.

For short-term prediction, the 1-year AUC of ALBI-SII was 0.686, exceeding that of Child–Pugh (0.624). Notably, the predictive advantage of ALBI-SII became more pronounced with longer follow-up. For 5-year OS prediction, the AUC of ALBI-SII reached 0.735, which was not only markedly higher than that of Child–Pugh (0.555) but also slightly exceeded that of CNLC staging (0.724). DeLong’s test confirmed no significant difference between the ALBI-SII Grade and CNLC staging (Z = 0.042, P = 0.967). These findings suggest that, for long-term survivors, hepatic reserve function and immune-inflammatory status may exert a greater influence on prognosis than initial tumor staging (**Figure 2**).

### 3.4 Nomogram construction and validation

**Figure 3:**
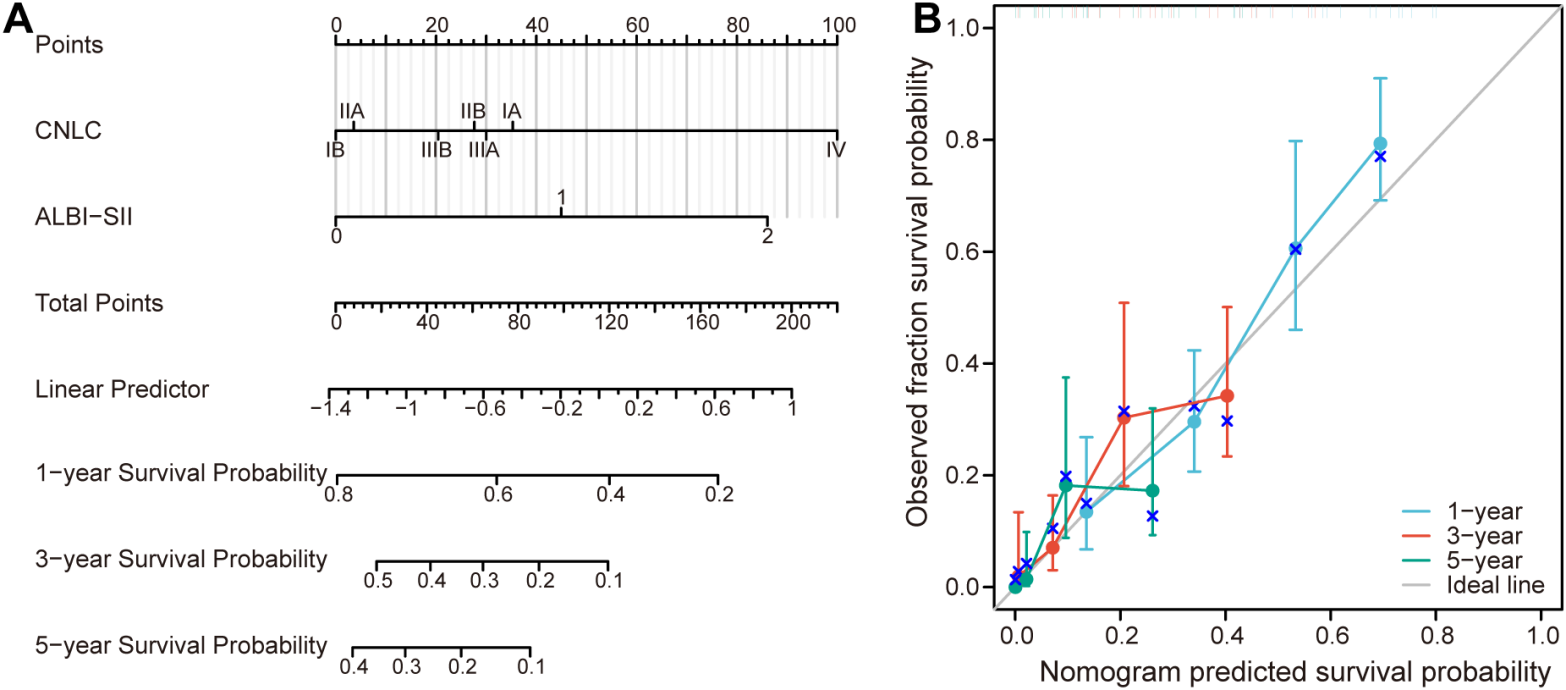
Nomogram construction and validation. **(A)** A prognostic nomogram integrating CNLC staging and ALBI-SII grade to predict 1-, 3-, and 5-year overall survival. To use the nomogram, locate the patient’s value on each variable axis, draw a vertical line upward to the “Points” axis to determine the score, sum the scores for “Total Points” and draw a vertical line downward to the survival axes to estimate the survival probability. **(B)** Calibration curves for 1-year (light blue), 3-year (orange), and 5-year (green) overall survival. The x-axis represents the nomogram-predicted survival probability, and the y-axis represents the observed actual survival fraction. The diagonal gray line represents the ideal prediction (perfect calibration). Vertical bars indicate 95% confidence intervals. ALBI, albumin-bilirubin; SII, systemic immune-inflammation index; CNLC, China Liver Cancer staging.

A nomogram integrating ALBI-SII grade and CNLC staging was constructed to predict 1-, 3-, and 5-year OS (**Figure 3A**). The specific point assignments for each predictor in the nomogram are detailed in **Table 3**. Although the CNLC staging showed marginal statistical significance in the multivariate Cox regression, its inclusion in the final nomogram increased the C-index from 0.703 to 0.858, confirming its essential role in improving the model’s prognostic accuracy. Calibration curves demonstrated excellent agreement between the predicted survival probabilities and the observed outcomes (**Figure 3B**).

**Table 3:** Nomogram scoring system for predicting overall survival.

Variables	Category	Points	Variables	Category	Points
ALBI-SII	0	0	CNLC	IIIA	65
	1	48		IIIB	82
	2	100		IV	96
CNLC	IA	0			
	IB	0			
	IIA	35			
	IIB	45			

The total risk score is calculated by summing the specific points assigned to each variable. Calculation: Total Score, Points (ALBI-SII Grade) + Points (CNLC Staging). Interpretation: A higher total score indicates a worse prognosis. ALBI, albumin-bilirubin; SII, systemic immune-inflammation index; CNLC, China Liver Cancer staging.

To rigorously assess the internal validity and stability of the prognostic model, a bootstrap resampling procedure with 5,000 iterations was performed. The multivariate model demonstrated robust discriminative ability, with an apparent Harrell’s C-index of 0.697 (95% CI: 0.663–0.732). The calculated optimism was negligible (0.002), yielding an optimism-corrected C-index of 0.695, which indicates that the model is not subject to severe overfitting and maintains high predictive accuracy. Furthermore, the bootstrap analysis confirmed the stability of the independent prognostic factors. The bootstrap-validated hazard ratios were 1.276 (95% CI: 1.172–1.416) for CNLC staging and 1.522 (95% CI: 1.202–1.970) for ALBI-SII grade. Both factors maintained their statistical significance and prognostic strength across the resampling cohorts, reinforcing their reliability as robust predictors for overall survival.

To further confirm the model’s stability across different time periods, we conducted a temporal validation analysis. The multivariate Cox model derived from the training cohort (N = 147) was applied to the independent temporal validation cohort (N = 63). Remarkably, the model achieved a Harrell’s C-index of 0.738 in the validation cohort. This performance was comparable to, and even slightly exceeded, that of the training cohort (C-index: 0.674), demonstrating the excellent robustness and generalizability of the ALBI-SII grade.

### 3.5 Risk score and clinical utility

**Figure 4:**
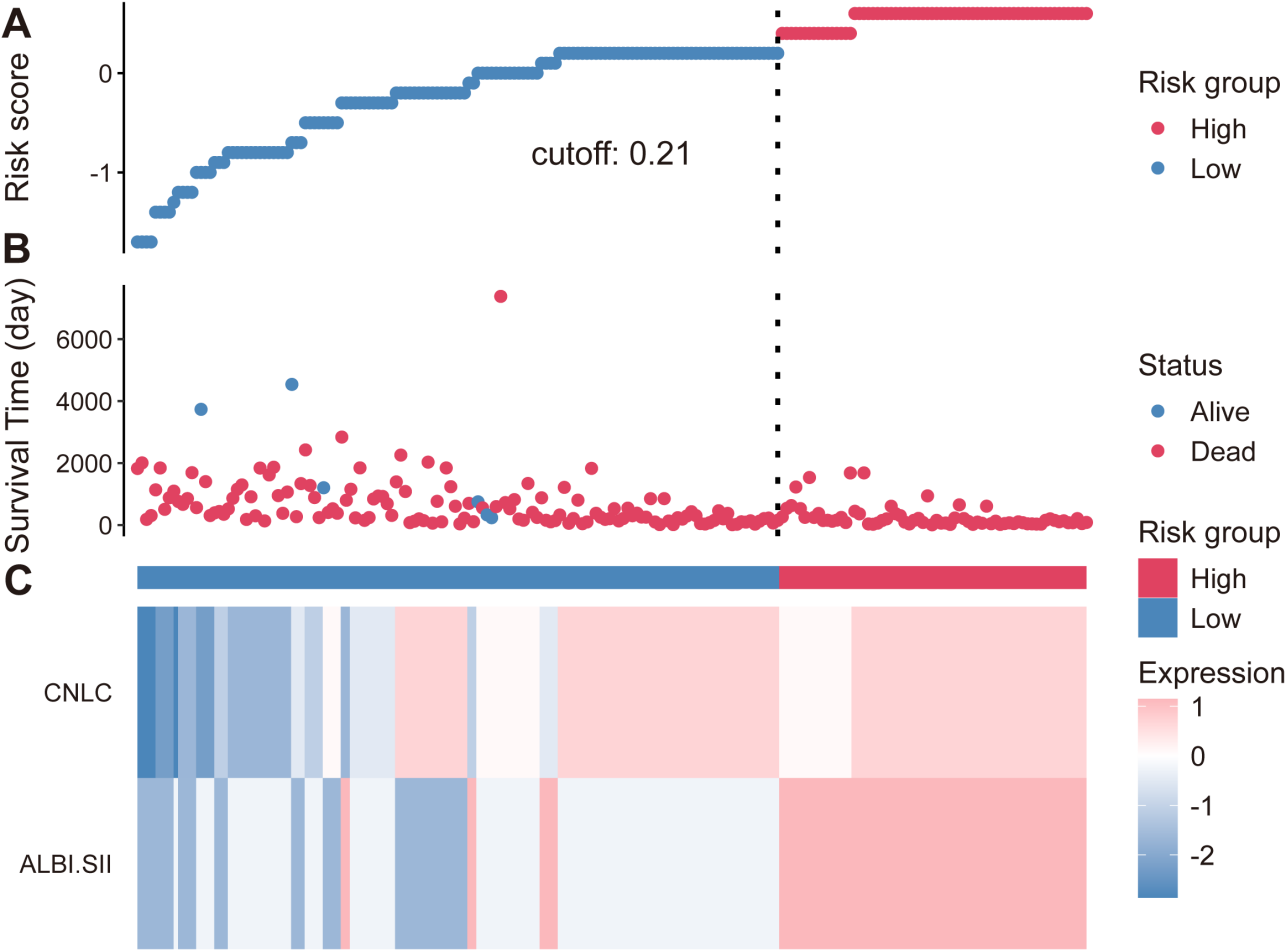
Risk score distribution and clinical characteristics. **(A)** Distribution of risk scores for the entire cohort. The dotted vertical line indicates the optimal cutoff value (0.21) separating patients into low-risk (blue dots) and high-risk (red dots) groups. **(B)** Survival status of patients corresponding to their risk scores. Red dots indicate deceased patients, while blue dots indicate surviving patients. **(C)** Heatmap showing the distribution of CNLC staging and ALBI-SII grades between the high-risk and low-risk groups. Colors represent the relative expression or grade level (blue, lower grade; red, higher grade). ALBI, albumin-bilirubin; SII, systemic immune-inflammation index; CNLC, China Liver Cancer staging.

A risk score was calculated based on the Cox regression coefficients. The risk score distribution plot visually demonstrated that increasing risk scores were associated with higher mortality density and shorter survival time. Heatmap analysis further revealed a close association between high risk scores, higher ALBI-SII grades, and advanced CNLC staging (**Figure 4**).

**Figure 5:**
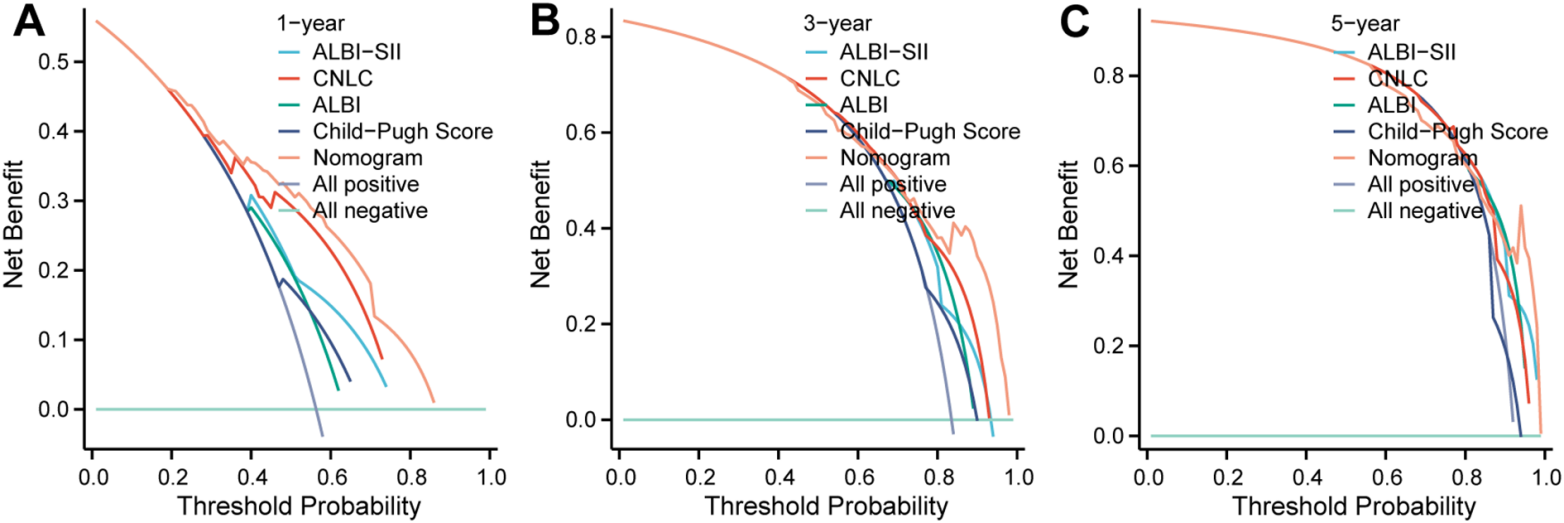
Decision curve analysis of prognostic models. The curves represent the net clinical benefit of the Nomogram (orange line), ALBI-SII grade (light blue line), CNLC staging (red line), ALBI grade (green line), and Child-Pugh score (dark blue line) for predicting **(A)** 1-year, **(B)** 3-year, and **(C)** 5-year overall survival. The y-axis measures the net benefit, while the x-axis displays the threshold probability. The horizontal teal line represents the assumption that no patients die (“All negative”), and the gray curve represents the assumption that all patients die (“All positive”). The nomogram consistently showed the highest net benefit across a wide range of threshold probabilities. DCA, Decision Curve Analysis; ALBI, albumin-bilirubin; SII, systemic immune-inflammation index; CNLC, China Liver Cancer staging.

Finally, DCA showed that the nomogram based on ALBI-SII provided greater net clinical benefit than the Child–Pugh classification or the “treat-all” or “treat-none” strategies across a wide range of threshold probabilities (**Figure 5**).

## 4 Discussion

In this study, we developed and validated a prognostic nomogram based on a novel ALBI-SII grade and CNLC staging for patients with HCC. Although the CNLC staging did not reach statistical significance in the multivariate analysis—likely due to the limited sample size and uneven distribution among subgroups—we incorporated it into the nomogram. Given that tumor burden (represented by CNLC) is a universally accepted cornerstone of HCC prognosis (2), its inclusion ensures the model’s clinical plausibility and allows for a more comprehensive assessment of patient survival by combining both tumor factors and host immune-liver status (ALBI-SII). The key finding is that ALBI-SII grade is not only an independent prognostic factor for OS but also demonstrates comparable predictive performance to the complex CNLC staging system, particularly for long-term survival, while being significantly simpler and more objective than the traditional Child–Pugh classification.

Recently, the combination of inflammation and liver function has gained increasing attention. Teng et al. (2023) reported that the ALBI-SII score could predict outcomes in patients with unresectable HCC receiving regorafenib sequential therapy (7). Consistent with their findings, our study confirmed the prognostic value of the ALBI-SII grade. However, our study expands the generalizability of this index in several key aspects. First, unlike Teng’s cohort which was limited to sorafenib-refractory patients, our study population included a real-world cohort across different CNLC staging (I–IV) receiving various treatments, including curative resection, ablation, and TACE. Second, we focused on long-term overall survival rather than just drug response, highlighting that ALBI-SII reflects the fundamental balance between tumor burden and host immune-nutritional status across the entire disease trajectory.

Several modifications of the ALBI grade have been proposed to enhance its prognostic value. For instance, the PALBI (Platelet-Albumin-Bilirubin) grade incorporates platelet counts to reflect the severity of portal hypertension (8), while the EZ-ALBI grade simplifies the calculation formula for easier clinical application (9). Although these indices have demonstrated utility, they have inherent limitations. PALBI primarily captures the hemodynamic consequences of cirrhosis (i.e., hypersplenism-induced thrombocytopenia) but overlooks the systemic inflammatory response, which is a hallmark of cancer progression. In contrast, the ALBI-SII grade offers a more comprehensive biological perspective. By integrating the SII, our model does not merely view platelets as a marker of portal hypertension but contextualizes them within the inflammatory network. The SII captures the interplay where platelets facilitate tumor cell arrest, neutrophils drive matrix remodeling, and lymphocytes exert antitumor cytotoxicity. Therefore, compared to PALBI or EZ-ALBI, the ALBI-SII grade is arguably superior because it simultaneously assesses three critical dimensions: hepatic metabolic reserve (ALBI), portal hypertension/coagulation status (Platelets), and the systemic immune-inflammatory microenvironment (Neutrophils and Lymphocytes). This multi-dimensional assessment explains why ALBI-SII outperformed standard staging systems in our cohort.

The prognostic robustness of the ALBI-SII grade is likely rooted in the complex interplay between liver dysfunction and the tumor immune microenvironment (TIME). The SII component serves as a surrogate marker for the balance between tumor-promoting inflammation and anti-tumor immunity. Specifically, beyond secreting vascular endothelial growth factor (VEGF) to promote angiogenesis, elevated neutrophils can release Neutrophil Extracellular Traps (NETs), which have been shown to trap circulating tumor cells and facilitate their adhesion to distant organs (10). Concurrently, platelets can induce Epithelial-Mesenchymal Transition (EMT) via the TGF-β/Smad signaling pathway and form a “protective cloak” around tumor cells, shielding them from Natural Killer (NK) cell-mediated lysis (11). Conversely, lymphopenia indicates a state of immune exhaustion and impaired T-cell surveillance. When combined with the ALBI grade—which reflects the physiological reserve of the liver (4)—the ALBI-SII grade essentially captures the relationship between the “soil” (liver background) and the “seed” (tumor aggressiveness). A high ALBI-SII grade implies a hypoxic, immunosuppressive, and inflammatory microenvironment that favors tumor progression and resistance to therapy.

An important finding of this study is the outstanding performance of ALBI-SII in long-term (5-year) survival prediction. The ROC analysis showed that the 5-year AUC of ALBI-SII was comparable to that of CNLC staging. A plausible biological explanation is that short-term survival is primarily driven by tumor burden (12). While ALBI-SII did not statistically outperform CNLC, this finding holds substantial clinical value. CNLC staging requires complex imaging assessments. which rely on subjective interpretation. In contrast, the ALBI-SII grade achieves an equivalent level of prognostic accuracy using only five objective, low-cost laboratory parameters. This suggests that ALBI-SII can serve as a simple, accessible surrogate marker for risk stratification, particularly in resource-limited settings or for rapid bedside assessment.

Beyond its prognostic utility, the ALBI-SII grade holds significant promise for guiding postoperative clinical decision-making, particularly regarding adjuvant therapies. Currently, the selection of candidates for adjuvant TACE or immune checkpoint inhibitors (ICIs) remains controversial. Our findings suggest that ALBI-SII could serve as a pragmatic stratification tool. For high-risk patients (Grade 2), who exhibit both high inflammatory burden and potentially compromised liver function, the risk of micrometastases escaping surgical resection is high. Therefore, this subgroup requires intensive postoperative surveillance and may derive the greatest benefit from aggressive adjuvant interventions to eliminate residual tumor cells. Conversely, for low-risk patients (Grade 0) who demonstrated excellent long-term survival, a “watch-and-wait” strategy may be sufficient, thereby avoiding overtreatment.

Furthermore, the robustness of our model was rigorously validated. To address potential selection bias caused by missing data, we performed a sensitivity analysis using multiple imputation, and the pooled results confirmed that ALBI-SII grade remained an independent prognostic factor. Additionally, multivariate analysis adjusted for treatment modalities (curative vs. non-curative) further verified that the prognostic value of ALBI-SII is independent of therapeutic interventions. The internal validation using 5,000 bootstrap resampling iterations and temporal validation supported the model’s stability. The visualization of risk score distribution and heatmaps enhanced model interpretability, while DCA confirmed its potential clinical utility in identifying high-risk patients who may benefit from intensified monitoring or intervention (13).

Several limitations should be acknowledged. First, this was a single-center retrospective study and may be subject to selection bias. Second, although rigorous internal validation and temporal validation were performed, external validation using independent multicenter cohorts is still required to confirm the generalizability of the model. Third, while we proposed potential mechanisms involving NETs and EMT, these biological pathways were not experimentally validated in this clinical study and warrant further basic research. Finally, the study period spanned from 2013 to 2021, a significantly long interval during which the standard of care for HCC evolved, particularly with the introduction of immune checkpoint inhibitors and targeted therapies. Although we adjusted for curative versus non-curative modalities, the specific impact of evolving systemic therapy regimens on the prognostic value of ALBI-SII could not be fully evaluated due to the limited sample size and the retrospective nature of the data.

## 5 Conclusions

In summary, the ALBI-SII grade is an objective, robust, and easily obtainable prognostic indicator. The nomogram integrating ALBI-SII and CNLC staging demonstrates excellent performance in predicting overall survival in patients with HCC, particularly for long-term survival, and significantly outperforms the traditional Child–Pugh classification. This model may serve as a valuable complement to CNLC staging, facilitating more precise risk stratification and individualized clinical decision-making.

## Data Availability

The original contributions presented in the study are included in the article/supplementary material. Further inquiries can be directed to the corresponding authors.
